# Characterization of complete mitochondrial genome of *Oreonectes furcocaudalis* (Cypriniformes, Balitoridae, Nemacheilidae)

**DOI:** 10.1080/23802359.2017.1280697

**Published:** 2017-02-15

**Authors:** Fuguang Luo, Jie Huang, Junling Liu, Ling Situ, Xia Liu, Yanhong Wen

**Affiliations:** Liuzhou Aquaculture Technology Extending Station, Liuzhou, China

**Keywords:** Mitochondrial genome, *Oreonectes furcocaudalis*, Nemacheilidae

## Abstract

*Oreonectes furcocaudalis* is a rare cave-dwelling loach that lives in the karst cave of Rongshui and Rong’an county, Southwestern China. In this study, the complete mitochondrial genome of *O. furcocaudalis* is sequenced using the Illumina Hiseq4000 platform with *de novo* strategy, with a circular molecule of 16,569 bp in size (GenBank accession number KX778472). It contains 13 protein-coding, 2 ribosomal RNA, 22 transfer RNA genes, and a 917 bp control region. Phylogenetic tree based on the complete mitogenome show that Oreonectes as a clade is close to the genus Lefua and Homatula.

*Oreonectes furcocaudalis*, a rare and endemic cave-dwelling loach, was first found in the karst cave of Rongshui county, Guangxi Province, Southwestern China (Zhu 1989; Du et al. 2008). So far, there are 10 effective species in this genus *Oreonectes*, and 8 of them distribute in Guangxi Province (Romero et al. 2009; Yang et al. 2011; Deng et al. 2016). However, the molecular and genetic research of this species was rare. In this study, we determined the complete mitochondrial genome of *O. furcocaudalis*, and analyzed the phylogenetic relationship of the loach in Nemacheilidae fishes, and expected to acquire useful information for studying on genetic status and evolutionary in this species.

In this study, the specimen was collected from subterranean river of Rong’an county (24°59′59″N, 109°28′02″E), Southwestern China in March, 2016, and it was stored in Liuzhou Aquaculture Technology Extending Station, Liuzhou, China. Then, we have sequenced the complete mitochondrial genome of *O. furcocaudalis* using the Illumina Hiseq4000 platform with *de novo* strategy, and submitted the genome into GenBank with accession number KX778472.

The complete mitochondrial genome of *O. furcocaudalis* was 16,569 bp in length, with the base composition of 31.03% A, 25.65% T, 27.39% C, 15.92% G, and an AT bias of 56.68%. This mitogenome also had a typical structure which consisted of 13 protein-coding, 2 ribosomal RNA (rRNA), 22 transfer RNA (tRNA) genes, and 1 control region (D-loop). All genes were encoded on the heavy strand but *ND6* gene and eight tRNA genes. Except for *COXI*, other protein-coding genes were initiated with the orthodox ATG. Meanwhile, the stop codons of the 13 protein-coding genes had four types (TAA, TAG, TA–, and T––). The length of all tRNA genes were ranged from 66 to 76 bp in size, and the 12S and 16S rRNA contained 957 bp and 1674 bp, respectively. A total of 917 bp control region (D-loop) was located between tRNA-Phe and tRNA-Pro. In all genes, the overlaps and spaces might be existed in adjacent gene, for example, the high level of overlaps and spaces was found in *tRNA-Asn* (30 bp spaces), *tRNA-Asp* (13 bp spaces), *ATP8* (10 bp overlaps), and *ND4L* (7 bp overlaps).

To investigate the phylogenetic position of *O. furcocaudalis* in Nemacheilidae fishes, a neighbour-joining(NJ) tree was constructed using MEGA 6.06. *O. furcocaudalis* was first clustered together with *O. platycephalus*, and this group was close to the genus Lefua and Homatula (Figure 1).

**Figure 1.**
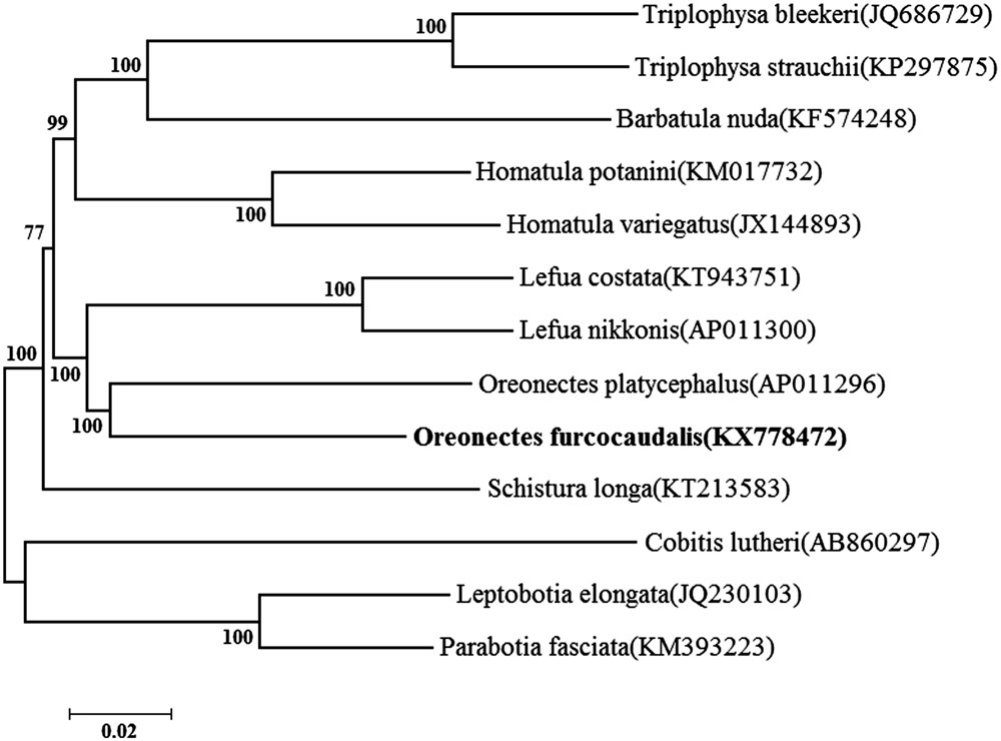
Neighbour-joining phylogenetic tree based on the mitochondrial genome of *O. furcocaudalis* and other 12 affinis fishes using MEGA 6.06. *Cobitis lutheri* served as an outgroup species.
